# CCTA-Guided Selective Invasive Coronary Catheterization: A Strategy to Reduce Contrast Volume and Improve Efficiency

**DOI:** 10.3390/diagnostics15070890

**Published:** 2025-04-01

**Authors:** Jorge Dahdal, Ruurt Jukema, Aernout G. Somsen, Eline Kooijman, Ellaha Wahedi, Jorrit S. Lemkes, Pieter G. Raijmakers, Ton Heestermans, Niels van Royen, Paul Knaapen, Ibrahim Danad

**Affiliations:** 1 Department of Cardiology, Amsterdam Cardiovascular Sciences, Amsterdam University Medical Centers, Vrije Universiteit Amsterdam, 1081 HV Amsterdam, The Netherlands; 2 Departamento de Enfermedades Cardiovasculares, Clínica Alemana de Santiago, Facultad de Medicina, Clínica Alemana Universidad Del Desarrollo, Santiago 7610658, Chile; 3 Cardiology Centre Netherlands, 1105 BJ Amsterdam, The Netherlands; 4 Amsterdam UMC—Location VUmc, Radiology, Nuclear Medicine & PET Research, 1081 HV Amsterdam, The Netherlands; 5 Department of Cardiology, Noordwest Ziekenhuisgroep, 1815 JD Alkmaar, The Netherlands; 6 Department of Cardiology, Radboud University Medical Center, 6525 GA Nijmegen, The Netherlands

**Keywords:** CCTA, FFR, CAD, ICA

## Abstract

**Background:** Symptomatic patients with unilateral obstructive coronary artery disease (CAD) identified by coronary computed tomography angiography (CCTA), involving either the right or left coronary artery, typically undergo per-protocol bilateral coronary visualization during invasive coronary angiography (ICA). However, a selective visualization approach may be sufficient. **Objectives:** The objectives of this study were to assess the accuracy of CCTA in excluding hemodynamically significant coronary stenosis in patients with unilateral CAD and to evaluate whether a CCTA-guided selective ICA strategy can reduce procedure time and contrast agent use. **Methods:** In this cross-sectional cohort study, 454 patients with clinically suspected stable CAD who underwent CCTA prior to ICA were included. The study population consisted of 190 patients with unilateral obstructive CAD, defined as ≥50% diameter stenosis on CCTA, and an absence of obstructive CAD on the contralateral side. ICA with invasive functional assessment was used as the reference standard. **Results:** CCTA demonstrated a high accuracy, 97.4% (95% CI: 94–99%), in excluding hemodynamically significant disease in the contralateral arteries without obstructive CAD. Compared to the conventional ICA approach, a CCTA-guided selective visualization strategy resulted in significant reductions in procedure time and contrast agent usage: procedure time and contrast agent usage were reduced by 27% (95% CI: 12.1–47.5%) and 46.8% (95% CI: 27.5–67.0%), respectively. **Conclusions:** In patients with unilateral obstructive CAD identified by CCTA, a CCTA-guided selective ICA visualization strategy is highly accurate in ruling out hemodynamically significant CAD on the contralateral side. Additionally, this unilateral ICA approach has the potential to reduce both contrast agent usage and procedure time compared to the conventional bilateral visualization strategy.

## 1. Introduction

Patients with suspected obstructive coronary artery disease (CAD) are commonly referred for invasive coronary angiography (ICA) to assess coronary artery anatomy and evaluate the need for revascularization. The standard ICA protocol includes bilateral visualization of both the left coronary artery (LCA) and the right coronary artery (RCA). Although diagnostic ICA carries a relatively low risk of complications, it exposes both patients and medical personnel to significant hazards and generates healthcare-related waste. In contrast, coronary computed tomography angiography (CCTA) provides a non-invasive, high-resolution visualization of the epicardial coronary arteries. Cumulative evidence supports that a CCTA-driven diagnostic strategy in patients with suspected CAD is not only safe but also cost-effective compared to traditional non-invasive approaches, resulting in its inclusion in various clinical guidelines as a primary diagnostic test [1,2,3,4]. Given CCTA’s strong ability to rule out CAD, recent studies have suggested that in patients with unilateral obstructive CAD (in either the LCA or RCA), a targeted unilateral visualization strategy focusing solely on the artery with CCTA-identified stenosis poses a low risk of missing significant coronary stenosis in the non-targeted artery [5,6]. This approach may also reduce radiation exposure and procedural times [5,6].

Recent initiatives from the World Health Organization advocate for more environmentally sustainable healthcare, emphasizing waste minimization and efficient resource management as key actions [7]. Despite this, initiatives aimed at reducing waste in the cardiac catheterization room have been limited, missing opportunities for significant reductions in operational costs and exposures [8]. Therefore, the primary objective of this study is to evaluate the accuracy of CCTA in excluding hemodynamically significant coronary stenoses of the non-obstructive coronary artery in patients with unilateral obstructive CAD, using ICA with invasive functional assessment as the reference standard. Additionally, we aim to assess whether a CCTA-guided unilateral visualization strategy affects the amount of contrast administered and the overall duration of the diagnostic procedure.

## 2. Methods

### 2.1. Design and Study Population

**Figure 1:**
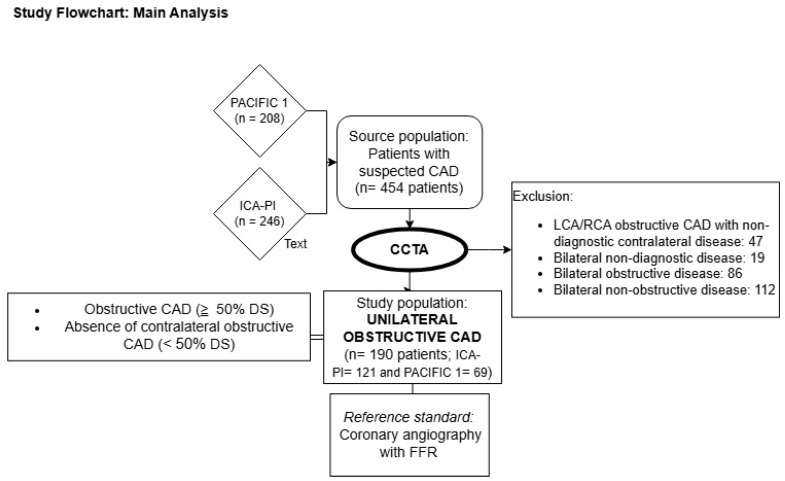
Study flowchart: main analysis. CAD: coronary artery disease, CCTA: coronary computed tomography angiography, FFR: fractional flow reserve, DS: diameter stenosis, LCA: left coronary artery, RCA: right coronary artery.

A cross-sectional study was conducted, combining participants from two cohorts as the study population. The Prospective Comparison of Cardiac PET/CT, SPECT/CT Perfusion Imaging, and CT Coronary Angiography With Invasive Coronary Angiography (PACIFIC 1) study comprises 208 patients with suspected CAD assessed by CCTA among other non-invasive imaging techniques prior to ICA between January 2012 and October 2014 [9]. The second cohort, the ICA-PI registry (NCT04815928), represents a clinical cohort in which all patients who underwent ICA on clinical indication were enrolled. Of patients enrolled in the ICA-PI registry, all patients with a CCTA prior to ICA were prospectively enrolled in the CT registry. This clinical registry included 246 patients who underwent CCTA between May 2020 and May 2023. Both investigations were conducted at Amsterdam UMC (VU University Medical Center, Amsterdam, the Netherlands). All patients underwent clinically indicated ICA within 90 days after the CCTA scan. Patients with renal insufficiency were excluded from both studies. Additionally, the PACIFIC 1 study excluded patients with prior CAD, ventricular arrhythmias, heart failure, or an estimated left ventricular ejection fraction (LVEF) < 50%. The ICA-PI CT registry has no official exclusion criteria. However, this patient population is driven by contemporary practice and therefore does not comprise patients with coronary stenting or arrhythmias precluding CCTA assessment. For the present study, the selected study population consisted of patients with unilateral obstructive CAD, defined as ≥50% diameter stenosis (DS) on CCTA, and no contralateral obstructive disease, defined as <50% DS. Figure 1 illustrates the study flowchart. Written informed consent was obtained from all participants. The ethics committee of Amsterdam UMC, location VU University Medical Center, approved the study protocols for PACIFIC 1 and ICA-PI. This research was conducted in accordance with the principles of the Declaration of Helsinki.

### 2.2. Coronary Computed Tomography Angiography

Patients underwent CCTA imaging using a 256-slice CT scanner (Philips Brilliance iCT, Best, The Netherlands) or a 192-slice dual-source CT scanner (Siemens Healthcare Somatom Force, Erlangen, Germany). Characteristics of the 256-slice scanner included a section collimation of 128 × 0.625 mm and a gantry rotation time of 270 ms, while the 192-slice dual-source scanner entailed a 192 × 0.6 mm section collimation and a gantry rotation time of 250 ms, respectively. Prior to CCTA, all the patients received 800 mcg of sublingual nitroglycerine. Prior to CCTA, heart rate (HR) was measured, and if necessary 50–150 mg of metoprolol was administered orally to lower the HR to a target of <65 bpm. If the HR remained >65 bpm during scanning an additional 5-to-25 mg of metoprolol was administered intravenously. Prospective ECG-gating was performed to reduce the radiation dose. A bolus of iodine contrast media was injected intravenously, followed by a saline chaser. The scan was triggered using an automatic bolus tracking technique, with the region of interest placed in the descending thoracic aorta and a threshold set at 150 HU.

The CCTA images were analyzed and interpreted by experienced radiologists blinded to the ICA results. The most severe stenosis in each major coronary artery was used for analysis. Following an intention-to-diagnose approach, noninterpretable segments with a diameter of >2.0 mm were considered positive for obstructive CAD. Patients with ≥1 coronary stenosis of ≥50% in the left main, circumflex, left anterior descending artery, or any of their major side branches were classified as having LCA obstructive CAD. Similarly, patients with ≥1 coronary stenosis of ≥50% in the RCA or its major branches were classified as having RCA obstructive CAD. Unilateral obstructive CAD, as identified by CCTA, was defined as the presence of obstructive CAD (≥50% DS) in either the RCA or LCA, with the absence of contralateral obstructive CAD. Additionally, a secondary definition for the absence of obstructive CAD was applied, characterized by an artery with no stenosis (0% DS) (see Supplementary Materials, Figure S1).

### 2.3. Invasive Coronary Angiography

ICA was conducted in accordance with our institutional protocol. Prior to the procedure, intracoronary administration of 0.2 mL of nitroglycerin was employed to induce coronary vasodilation. At least two orthogonal views were obtained per evaluated coronary artery. In the PACIFIC 1 study, routine intracoronary pressure measurements of the major coronary arteries were performed. In the ICA-PI study, major coronary arteries identified as having lesions with a DS ≥ 30% underwent intracoronary pressure assessment. Pressure wire measurements were avoided in subtotal lesions due to the risk of wire perforation. These lesions, including totally occluded arteries, were deemed hemodynamically significant. Aortic and intracoronary pressure measurements were conducted using a 0.014-inch sensor-tipped pressure wire (ComboWire, Volcano Corporation, Rancho Cordova, CA, USA, or PressureWire X, Abbott Vascular, Santa Clara, CA, USA) during pharmacologically induced hyperemia achieved with either intravenous (140 mcg/kg/min) or intracoronary adenosine (150 mcg). Hemodynamically significant CAD was defined as a fractional flow reserve (FFR) ≤ 0.80.

### 2.4. Procedural Characteristics of the Unilateral ICA Visualization Strategy

From the ICA-PI registry, data on the duration and contrast volume used for the diagnostic assessment of the LCA and RCA during ICA were prospectively collected for consecutively included patients. This consecutive sample was chosen at random to avoid any bias. This included key components of the diagnostic process including the time required for catheter changes, coronary artery engagement, image acquisition, and invasive hemodynamic measurements. If a PCI was performed during the index ICA, the time allocated to the interventional procedure was excluded from the diagnostic process. For each patient, the diagnostic procedural time and contrast volume were calculated and reported for both the conventional bilateral approach and the CCTA-guided unilateral visualization strategy. A detailed case example of these calculations is provided in the Supplementary Materials (Figure S2).

### 2.5. Statistical Analysis

Categorical variables are presented as numbers and percentages, while continuous variables are reported as means with standard deviations (SDs) or medians with interquartile ranges (IQRs), depending on their distribution. This study focused on evaluating the CCTA’s performance to categorize patients with unilateral obstructive CAD. The primary analysis focused on determining CCTA’s accuracy in ruling out hemodynamically significant CAD, quantified by its negative predictive value (NPV), defined as the proportion of true negatives (TNs) among all negative test results: NPV = TNs/(TNs + false negatives (FNs)). TNs and FNs with their demographic, clinical, and image features were reported and compared using Welch’s *t*-test or Fisher’s Exact Test as appropriate. The secondary analysis involved comparison of the procedure time and contrast volume used during the diagnostic ICA between the conventional bilateral approach and the CCTA-guided unilateral visualization strategy. The paired samples *t*-test was used to compare procedural times and contrast volumes between the two strategies. A two-sided *p*-value < 0.05 was considered statistically significant. All statistical analyses were performed using SPSS Statistics (IBM, version 27, Armonk, NY, USA) and R Studio (R Foundation for Statistical Computing, Version 4.2.1, Vienna, Austria).

## 3. Results

A total of 454 participants with suspected CAD were screened for inclusion, of whom 190 (41.9%) were identified with unilateral obstructive CAD by CCTA. Among them, 121 patients (63.7%) were from the ICA-IPI cohort, while 69 patients (36.3%) were from the PACIFIC 1 cohort. The study population had a mean age of 61 ± 9 years and consisted of 68% men. Hypertension was highly prevalent, affecting 48% of the study subjects (Table 1).

Regarding the CCTA findings, the RCA was identified as the non-obstructive artery in 83.7% (95% CI: 78–89%) of patients. Additional demographic, clinical, and imaging characteristics are presented in Table 1.

### 3.1. CCTA’s Performance in Excluding Significant CAD

CCTA demonstrated an accuracy of 97.4% (95% CI: 94–99%) for excluding hemodynamically significant CAD (Table 2). An accuracy of 96.7% (95% CI: 91.8–98.7) was observed in the ICA-IPI cohort and an accuracy of 98.6% (95% CI: 92.2–99.7) was observed in the PACIFIC 1 cohort. Five patients were classified as FN, accounting for 2.6% (95% CI: 1–6%) of the study population.

Table 3 outlines the differences in clinical characteristics between the FN and TN groups. Notably, BMI was higher in the FN group (29 ± 9) compared to the TN group (26 ± 4), although this difference did not reach statistical significance (*p* = 0.08). There was no significant difference in the DS% of the arteries classified as non-obstructive by CCTA between the TN and FN groups (*p* = 0.19). However, the prevalence of 1–24% stenosis in arteries without obstructive CAD on CCTA was numerically higher in the FN group compared to the TN group (80% vs. 43%, Table 3). Among the five patients who had hemodynamically significant CAD despite CCTA indicating the absence of obstructive CAD, only two underwent revascularization. The remaining three FN cases had predominantly distal lesions or stenoses in small coronary arteries, which were deemed unsuitable for revascularization by the treating interventional cardiologists. Further details regarding the imaging characteristics and management of each FN case are provided in the Supplementary Materials (Table S1 and Figures S3–S7).

The accuracy of CCTA in excluding significant CAD, as strictly defined using FFR or ICA as the reference standard (98.3% [95% CI: 94–100] and 93.7% [95% CI: 89–96], respectively), is presented in the Supplementary Materials (Tables S2 and S3).

When employing a stricter definition for the absence of obstructive CAD on CCTA (DS% = 0), 64 out of 454 patients (14.9%) met the criteria for unilateral CAD by CCTA, as shown in the Supplementary Materials (Figure S1). Using this definition, the accuracy of CCTA for excluding hemodynamically significant CAD was 100% (95% CI: 94–100%) (Table 4).

### 3.2. Procedural Time and Contrast Use Following a CCTA-Guided Unilateral Visualization Strategy

**Figure 2:**
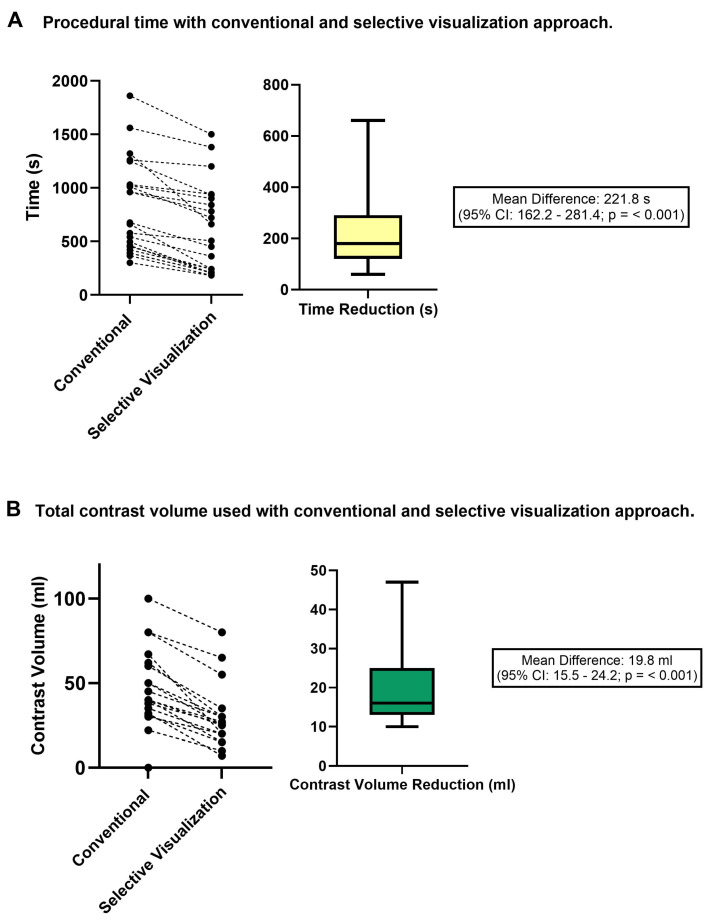
Differences in procedural times (**A**) and contrast administration (**B**) following a conventional bilateral catheterization approach and a CCTA-guided unilateral visualization ICA approach. CCTA: coronary computed tomography angiography; ICA: invasive coronary angiography.

The demographic and clinical characteristics of the patient subset with the available data on procedure time and contrast administration (*n* = 22) did not significantly differ from those of the study population (Supplementary Materials, Table S4). The implementation of a unilateral visualization strategy reduced the diagnostic angiography procedure time by 221.8 s (95% CI: 162.2–281.4), corresponding to a 27% relative decrease (95% CI: 12.1–47.5%, *p* < 0;01) compared to the conventional bilateral diagnostic strategy (Figure 2A). In terms of contrast administration, the unilateral visualization strategy resulted in a reduction of 19.8 mL (95% CI: 15.5–24.2), equivalent to a 46.8% relative decrease (95% CI: 27.5–67.0%, *p* < 0.01) in contrast volume during ICA compared to the conventional catheterization approach (Figure 2B).

## 4. Discussion

The main findings of this study can be summarized as follows: In patients with CCTA-defined unilateral obstructive disease, the presence of hemodynamically obstructive CAD in the contralateral artery was accurately excluded by CCTA. Furthermore, compared to a conventional bilateral coronary angiography approach, the use of a CCTA-guided selective visualization strategy may result in significant reductions in ICA procedure times and contrast administration.

In recent years, major international guidelines and professional societies have endorsed CCTA as the preferred non-invasive diagnostic test for evaluating patients with suspected chronic coronary syndrome. This endorsement has led to a substantial increase in the use of CCTA across various regions globally [10,11]. Indeed, data from a recent multinational registry have shown that CCTA was selected as the initial diagnostic test in approximately one-fifth of patients with suspected stable CAD [12]. It is important to highlight that in our clinical cohort obstructive CAD limited to either the left or right coronary artery was observed in over one-third of patients, a finding consistent with prior reports [5]. Therefore, considering the significant annual volume of CCTA scans conducted worldwide, it is reasonable to infer that identifying patients with unilateral obstructive CAD by CCTA represents a common clinical scenario among those patients referred for ICA [13,14].

As we have demonstrated, given the high rule-out power of CCTA, an invasive selective visualization strategy guided by scan results has a low probability of overlooking significant CAD. Echoing our findings, an observational study by van Beek et al., which included 202 patients with unilateral obstructive CAD on CCTA, demonstrated that this strategy has a 99.5% accuracy for excluding angiographically significant coronary stenoses [5]. Unlike the study by van Beek et al., a key strength of the present study is the use of FFR measurements to discern the hemodynamic significance of CAD, which has shown superior outcomes in guiding revascularization [15]. It is crucial to emphasize that less than half of the patients with hemodynamically significant CAD who were misdiagnosed by CCTA ultimately underwent revascularization. The remaining patients with false-negative CCTA results had coronary stenoses located either too distally or in small coronary arteries. Recent cumulative evidence from randomized clinical trials have shown that revascularization in chronic coronary syndromes does not prevent death or myocardial infarction when added to guideline-directed medical therapy [16,17,18]. Since the proposed unilateral visualization strategy is conducted in patients with documented CAD as identified by CCTA, the recognition of the presence of atherosclerosis should prompt the early initiation of guideline-directed medical therapy, which remains the cornerstone of CAD management.

The possibility of circumventing unnecessary steps during a diagnostic ICA could potentially reduce the uncommon but non-negligible complications associated with diagnostic catheter advancement and coronary artery engagement. Additionally, it may minimize adverse effects related to the use of contrast agents, such as contrast-induced nephropathy [19,20]. Beyond the well-established clinical risk factors for developing contrast-induced nephropathy, procedural factors such as the total contrast and recent exposure to contrast agents are also associated with an increased risk of this adverse event [21,22]. According to our findings, the adoption of a unilateral visualization strategy could reduce the use of contrast usage by approximately 20 mL, which corresponds to a relative reduction of nearly 50% in contrast volume used during a diagnostic procedure. Furthermore, the implementation of this approach may enhance the efficiency and sustainability of our clinical process. Notably, it can considerably shorten the duration of the diagnostic procedure, with a relative reduction of 27% (95% CI: 12.1–47.5%). Additionally, this protocol may also eliminate the need for unnecessary diagnostic catheters. Considering the prevailing market prices of diagnostic coronary catheters, set between 10 and 30 EU, and the large volumes of procedures performed worldwide, the long-term and global economic impact of this simple workflow adjustment could be substantial. Recent studies have shown that the average waste generated per procedure in the catheterization laboratory is approximately 0.72 kg, amounting to an estimated total of 50 kg of recyclable waste accumulated weekly in a single academic center [23]. Thus, from a sustainability-oriented perspective, it is imperative to educate and empower medical teams in the catheterization room about the judicious use of resources and the implementation of effective waste management strategies.

Left-sided cardiac catheterization exposes both patients and healthcare personnel to significant levels of radiation, with average effective doses for a diagnostic procedure reported between 2 and 20 mSV [24]. Interventional cardiologists, among healthcare professionals, encounter the highest cumulative radiation exposure, thereby increasing their risk of long-term radiation-related diseases, including malignancies, skin lesions, orthopedic disorders, and cataracts [25,26]. No level of ionizing radiation is considered entirely risk-free, regardless of its magnitude. Furthermore, repeated exposure to ionizing radiation amplifies the risk, which correlates directly with the cumulative dose [27]. Recognizing these risks, there is an urgent need to explore strategies to minimize the exposure of healthcare workers to radiation [28]. Established radiation safety guidelines advocate for radiation doses to be “as low as reasonably achievable”, with consistent monitoring to ensure exposure does not exceed the recommended annual or lifetime thresholds [29]. Albeit not explored in this study, previous studies have shown that adopting an ultra-selective catheterization approach could be an effective strategy to significantly decrease the effective radiation exposure [5,6]. While the impact may appear marginal on an individual procedure basis, implementing this strategy on a large scale could lead to significant reductions in the cumulative radiation healthcare personnel are exposed to in the long term.

## 5. Limitations

The primary limitation of this study is the absence of clinical outcome data. However, CCTA is already utilized as a gatekeeper for ICA, and our study further confirms CCTA’s high performance in excluding obstructive CAD. Additionally, the modest benefit on prognosis in contemporary revascularization trials indirectly supports the notion that a unilateral visualization strategy may be a potentially safe approach. Secondly, procedure time and contrast volume were only prospectively recorded for a subset of the study population. Therefore, estimates of time and contrast savings should be interpreted as general references rather than precise measurements. Third, calculating the radiation dose separately for the LCA and RCA was technically unfeasible. However, in light of existing evidence we can hypothesize that adopting a unilateral visualization strategy will likely result in reduced radiation exposure [5,6]. Fourth, a dedicated cost-effectiveness assessment should be performed in the future to evaluate the impact of applying this approach on a global scale.

## 6. Conclusions

In patients with unilateral obstructive CAD identified by CCTA, a CCTA-guided unilateral ICA visualization strategy focusing only on the coronary artery with obstructive CAD demonstrated high accuracy in excluding hemodynamically significant CAD on the contralateral side. Furthermore, a unilateral ICA visualization strategy has the potential to reduce contrast usage, procedure time, and radiation dose compared to a conventional bilateral visualization strategy.

## Figures and Tables

**Table 1:** Demographic, clinical, and CCTA characteristics of patients classified as having unilateral disease by CCTA (*n* = 190 patients).

Variables	
Age in years (mean ± SD)	61 ± 9
Male sex (%)	129 (68%)
Diabetes mellitus (%)	28 (15%)
Hypertension (%)	91 (48%)
Hypercholesterolemia (%)	69 (35%)
Current smoker (%)	21 (11%)
Previous CAD (%)	3 (1.6%)
BMI (mean ± SD)	26.4 ± 4
CCTA features	
Obstructive CAD side DS%	
50–69%	50 (26%)
70–99%	87 (46%)
100%	10 (5%)
Non-diagnostic	43 (23%)
Non-obstructive CAD side DS%	
0%	64 (34%)
1–24%	84 (44%)
25–49%	42 (22%)

CCTA: coronary computed tomography angiography, CAD: coronary artery disease, BMI: body mass index, DS: diameter stenosis.

**Table 2:** Diagnostic accuracy of CCTA for excluding hemodynamically significant CAD using the primary CCTA definition for an absence of obstructive CAD (<50% DS) (*n* = 190 patients).

		Absence of Hemodynamically Significant CAD	Hemodynamically Significant CAD
CCTA	Absence of Obstructive CAD (<50% DS)	185 (97.4%, 95% CI: 94–99)	5 (2.6%, 95% CI: 1–6)
		True Negatives	False Negatives

CCTA: coronary computed tomography angiography, CAD: coronary artery disease, DS: diameter stenosis.

**Table 3:** Characteristics of true negatives (*n* = 185) and false negatives (*n* = 5).

	True Negative (*n* = 185 Patients)	False Negative (*n* = 5 Patients)	*p*-Value
Age in years (mean ± SD)	60.5 ± 9	62.2 ± 12	0.69
Male sex (%)	122 (66%)	5 (100%)	0.11
Diabetes mellitus (%)	27 (32%)	1 (20%)	0.93
Hypertension (%)	87 (46%)	4 (80%)	0.35
Hypercholesterolemia (%)	61 (37%)	4 (80%)	0.72
Smoking history (%)	21 (11%)	0 (0%)	0.51
Previous CAD (%)	3 (2%)	0 (0%)	0.77
BMI (mean ± SD)	26 ± 4	29 ± 9	0.08
CCTA characteristics			
Artery with non-obstructive CAD			
RCA (%)	154 (83%)	5 (100%)	0.32
LCA (%)	31 (17%)	0 (0%)	
Artery with non-obstructive CAD			
25–49 DS%	41 (22%)	1 (20%)	0.19
1–24 DS%	80 (43%)	4 (80%)	
0 DS%	64 (35%)	0 (0%)	
Invasive measurements			
FFR (mean ± SD)	0.94 ± 0.06	0.61	<0.01

CCTA: coronary computed tomography angiography, CAD: coronary artery disease, BMI: body mass index, RCA: right coronary artery, DS: diameter stenosis, FFR: fractional flow reserve.

**Table 4:** Diagnostic accuracy of CCTA for excluding hemodynamically significant CAD using a secondary CCTA definition for an absence of obstructive CAD (DS = 0%) (*n* = 64 patients).

		Absence of Hemodynamically Significant CAD	Hemodynamically Significant CAD
CCTA	Absence of Obstructive CAD (0% DS)	64 (100%, 95% CI: 94–100)	0 (0%, 95% CI: 0–6)
		True Negatives	False Negatives

CCTA: coronary computed tomography angiography, CAD: coronary artery disease, DS: diameter stenosis.

## Data Availability

Data available on request.

## Supplementary Materials

The following supporting information can be downloaded at: https://www.mdpi.com/article/10.3390/diagnostics15070890/s1, Figure S1: Study workflow using the secondary CCTA definition for absence of obstructive CAD (0% DS); Figure S2: Case example: Calculation of procedure time and contrast administration on both ICA approaches; Figure S3: Patient 1; Figure S4: Patient 2; Figure S5: Patient 3; Figure S6: Patient 4; Figure S7: Patient 5; Table S1: Characteristics and management of patients misclassified by CCTA (False Negatives); Table S2: Diagnostic accuracy of CCTA for excluding hemodynamic significant CAD. Using only FFR as reference standard (*n* = 115 of 190 patients); Table S3: Diagnostic accuracy of CCTA for excluding significant CAD. Using only ICA as reference standard (*n* = 190 patients); Table S4: Difference in demographic and clinical characteristics in the total study population and the group where procedure time and contrast use was measured.

## Abbreviations

CAD	Coronary Artery Disease
ICA	Invasive Coronary Angiography
LCA	Left Coronary Artery
RCA	Right Coronary Artery
CCTA	Coronary Computed Tomography Angiography
DS	Diameter Stenosis
FFR	Fractional Flow Reserve
TN	True Negative
FN	False Negative
PCI	Coronary Percutaneous Intervention
