# Supplementary material for: CCTA-Guided Selective Invasive Coronary Catheterization: A Strategy to Reduce Contrast Volume and Improve Efficiency
Source: Diagnostics (Basel). 2025 Apr 1;15(7):890. doi: 10.3390/diagnostics15070890 (PMC11988798; doi:10.3390/diagnostics15070890)

Supplementary Material:

Table S1. Characteristics and management of patients misclassified by CCTA (False Negatives).

**Patient**	**CCTA results**	**ICA/FFR**	**Management**
**1 (Figure S3)**	Multiple mixed plaques in middle and distal RCA, minimal stenosis (1-24% DS)	Distal RCA and RDP with > 70% DS lesions<br>FFR 0.61	3 vessels disease, CABG performed.
**2**<br>**(Figure S4)**	Mixed plaques in middle and distal RCA, minimal stenosis (1-24% DS)	Middle RDP2 > 90% DS lesion	Medical therapy indicated.
**3**<br>**(Figure S5)**	Small RCA, multiple calcified plaques diffusely distributed along the RCA, minimal stenosis (1-24% DS)	Distal RDP with > 90% DS lesion	No revascularization. Medical therapy indicated.
**4**<br>**(Figure S6)**	Multiple calcified plaques diffusely distributed along the RCA, minimal stenosis (1-24% DS)	Distal RPD with > 90% DS lesion	PCI RPD
**5**<br>**(Figure S7)**	Small RCA, multiple calcified plaques diffusely distributed along the RCA, minimal stenosis (1-24% DS)	CTO in distal RCA	No revascularization. Medical therapy indicated.

CAD: Coronary Artery Disease, CCTA: Coronary Computed Tomography Angiography, RDP: Right Descending Posterior, RCA: Right Coronary Artery, FFR: Fractional Flow Reserve , DS: Diameter Stenosis. PCI: Percutaneous Coronary Intervention.

Table S2. Diagnostic accuracy of CCTA for excluding hemodynamic significant CAD. Using only FFR as reference standard (n= 115 of 190 patients).

		**Absence of hemodynamically significant CAD (FFR > 0.8)**	**Hemodynamically significant CAD (FFR ≤ 0.8)**
CCTA	**Absence of obstructive CAD** (< 50% DS)	113 (98.3%, 95% CI: 94-100)	2 (1.7%, 95% CI: 1-6)
		**True Negatives**	**False Negatives**

FFR: Fractional Flow Reserve, CCTA: Coronary Computed Tomography Angiography, CAD: Coronary Artery Disease. DS: Diameter Stenosis.

Table S3. Diagnostic accuracy of CCTA for excluding significant CAD. Using only ICA as reference standard (n= 190 patients).

		Absence of significant CAD (< 50% DS)	Significant CAD (>50% DS)
CCTA	**Absence of obstructive CAD** (< 50% DS)	178 (93.7%, 95% CI: 89-96)	12 (6.3%, 95% CI: 4-11)
		**True Negatives**	**False Negatives**

FFR: Fractional Flow Reserve, CCTA: Coronary Computed Tomography Angiography, CAD: Coronary Artery Disease. DS: Diameter Stenosis.

Table S4. Difference in demographic and clinical characteristics in the total study population and the group where procedure time and contrast use was measured.

**Variables**	**Study Population (n = 190 patients)**	**Time and Contrast measured (n = 22 patients)**	p value
**Age in years (Mean ± SD)**	61 ± 9	62.1 ± 5.7	0.61
**Male Sex (%)**	129 (68%)	16 (73%)	0.63
**Diabetes Mellitus (%)**	28 (15%)	2 (9%)	0.44
**Hypertension (%)**	91 (48%)	11 (50%)	0.86
**Hypercholesterolemia (%)**	69 (36%)	10 (45%)	0.41
**Current smoker (%)**	21 (11%)	4 (18%)	0.34
**Previous CAD (%)**	3 (1.6%)	1 (5%)	0.27
**BMI (Mean ± SD)**	26.4 ± 4	27.1 ± 4.1	0.44

CCTA: Coronary Computed Tomography Angiography, CAD: Coronary Artery Disease. BMI: Body Mass Index, RCA: Right Coronary Artery, DS: Diameter Stenosis, FFR: Fractional Flow Reserve.

Figure S1. Study workflow using the secondary CCTA definition for absence of obstructive CAD (0% DS).

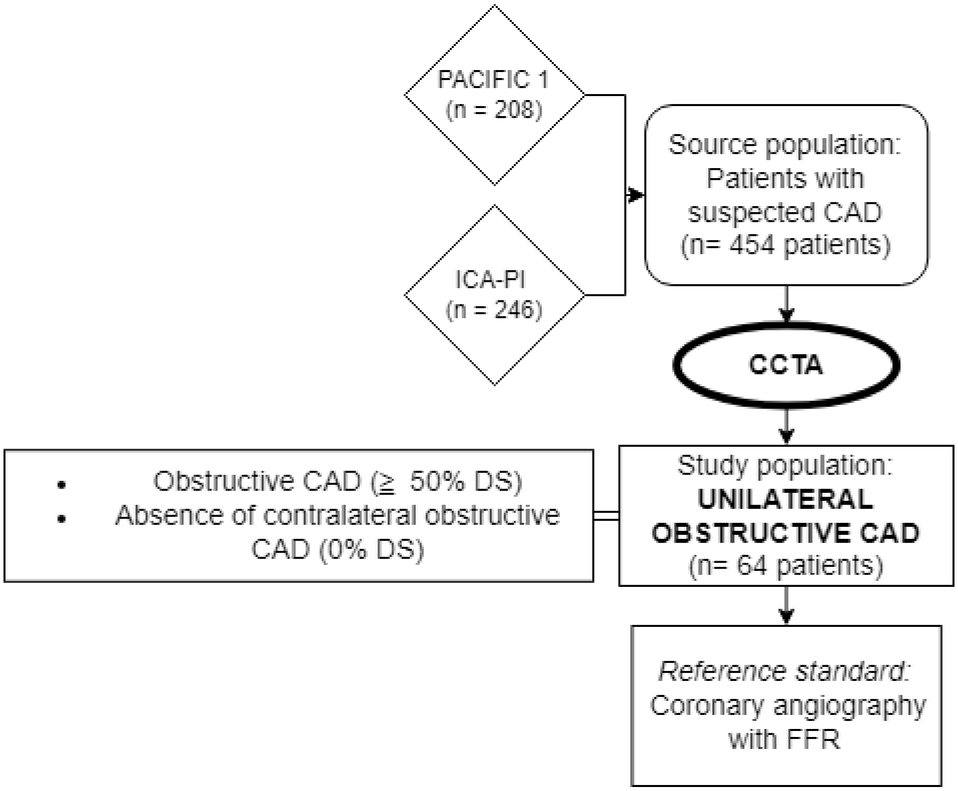

Study workflow using secondary definition of absence of obstructive CAD by CCTA. *CAD: Coronary Artery Disease, CCTA: Coronary Computed Tomography Angiography, FFR: Fractional Flow Reserve, DS: Diameter Stenosis.*

Figure S2 . Case example: Calculation of procedure time and contrast administration on both ICA approaches.

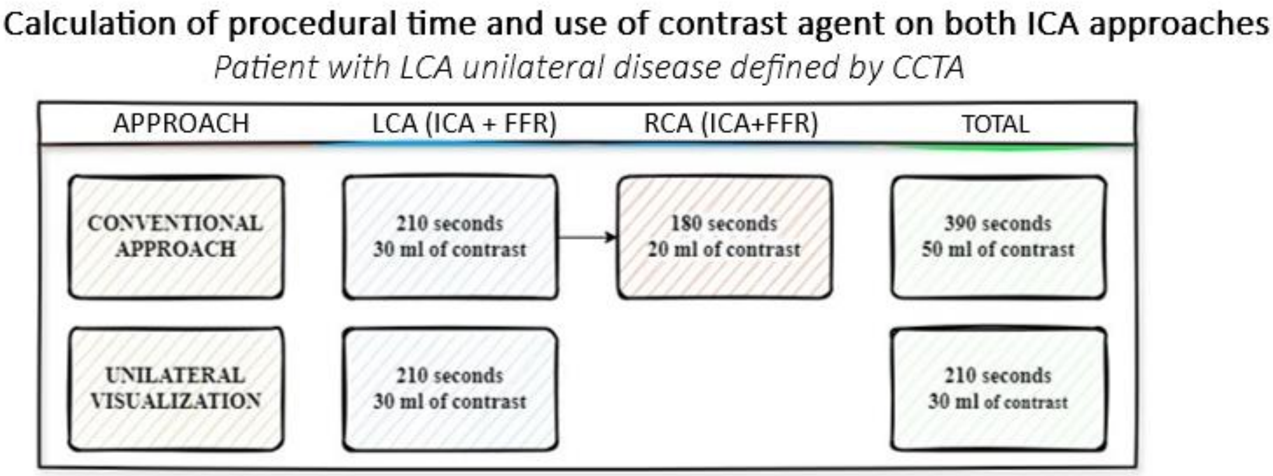

Diagnostic procedural time (s) and use of contrast agent (ml) was prospectively collected for a subpopulation of patients (n= 22). If PCI was performed during the index procedure this was not included as part of the diagnostic procedure. The CCTA-guided selective unilateral visualization approach diagnostic procedure time and contrast administration were computed retrospectively. *CCTA: Coronary Computed Tomography Angiography, ICA: Invasive Coronary Angiography, FFR: Fractional Flow Reserve, DS: Diameter Stenosis.*

Figure S3. Patient 1

Patient 1

CCTA	ICA
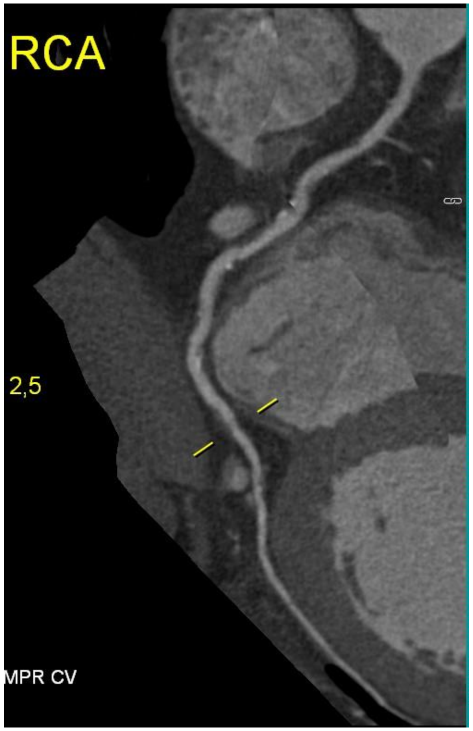	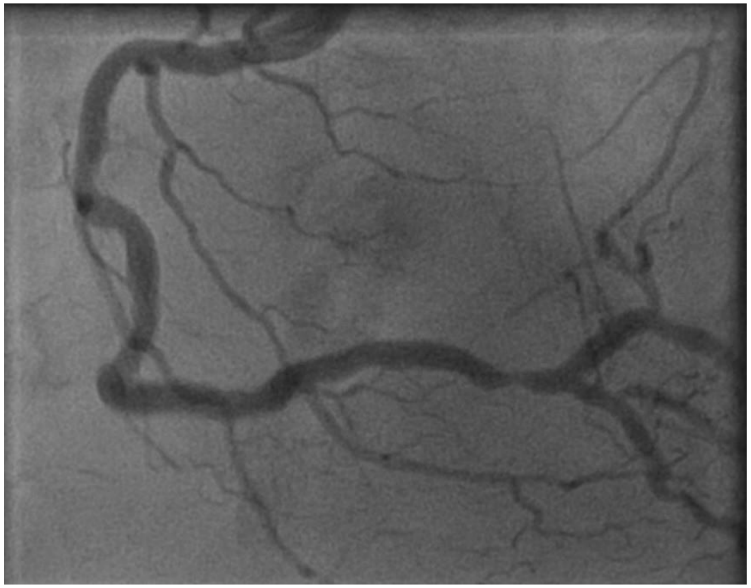

Figure S4 Patient 2

Patient 2

CCTA	ICA
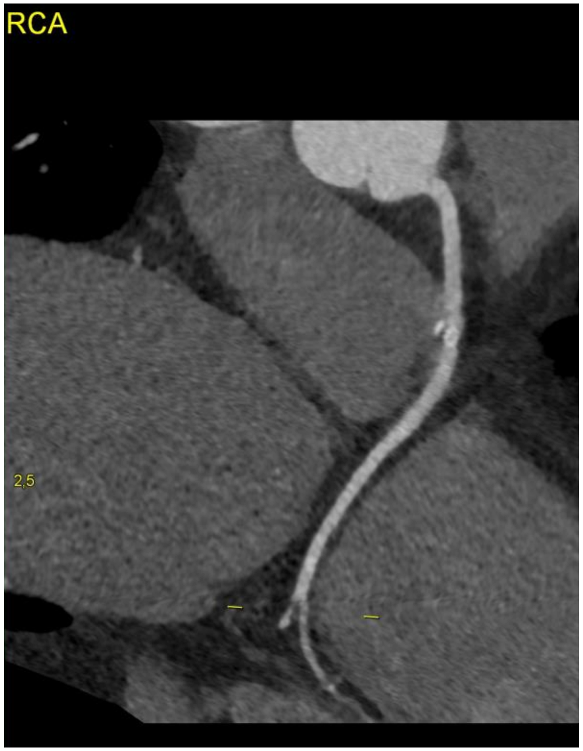	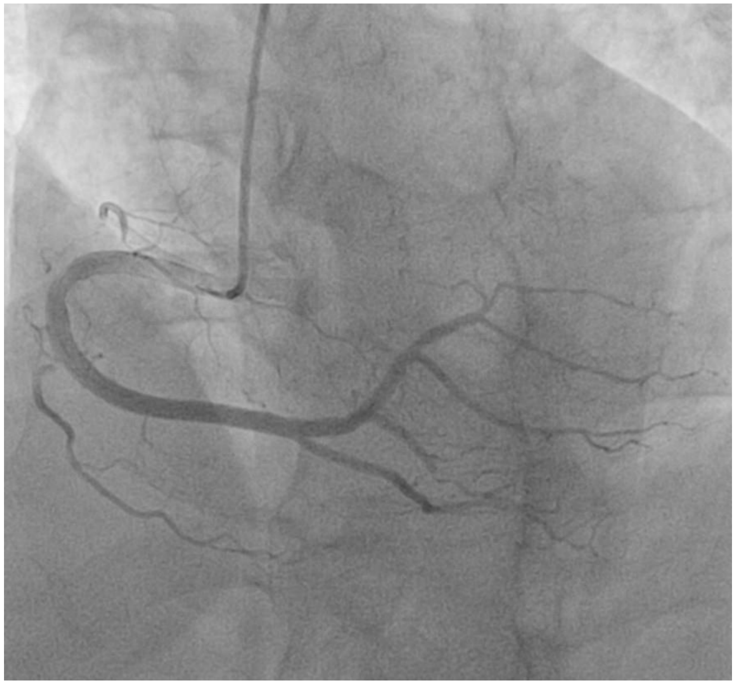

Figure S5 Patient 3

Patient 3

CCTA	ICA

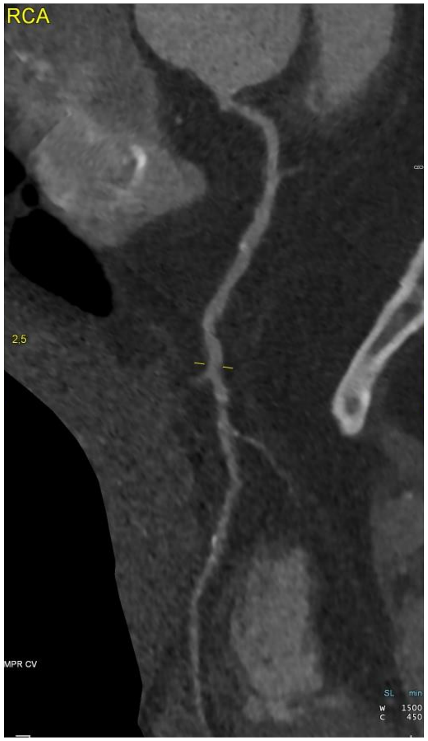

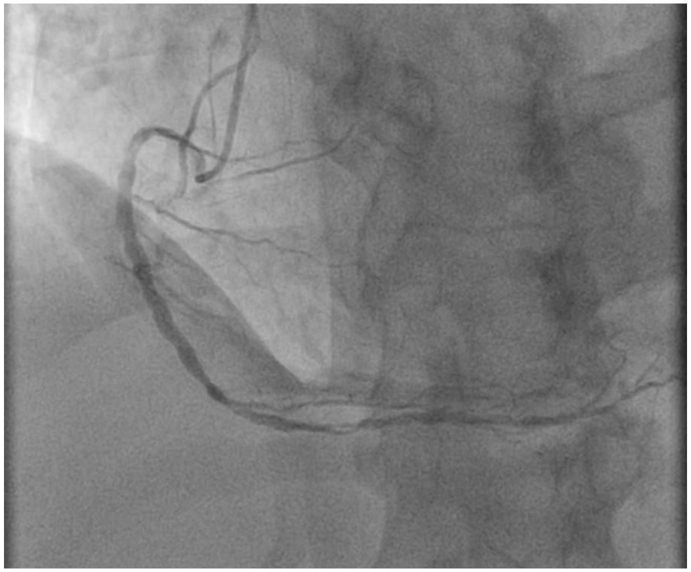

Figure S6 Patient 4

Patient 4

CCTA	ICA

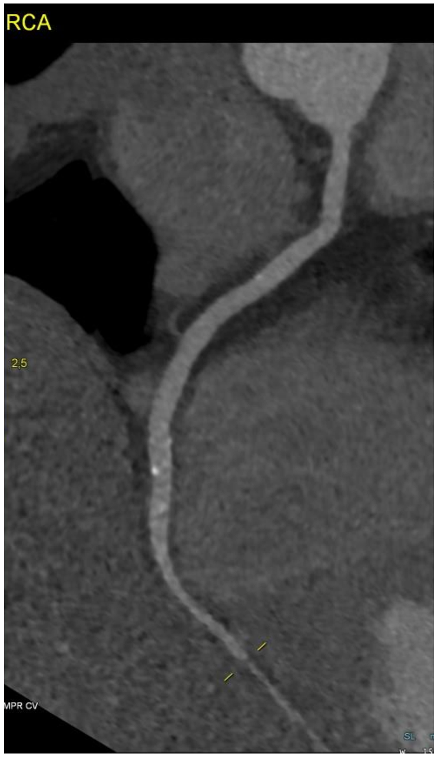

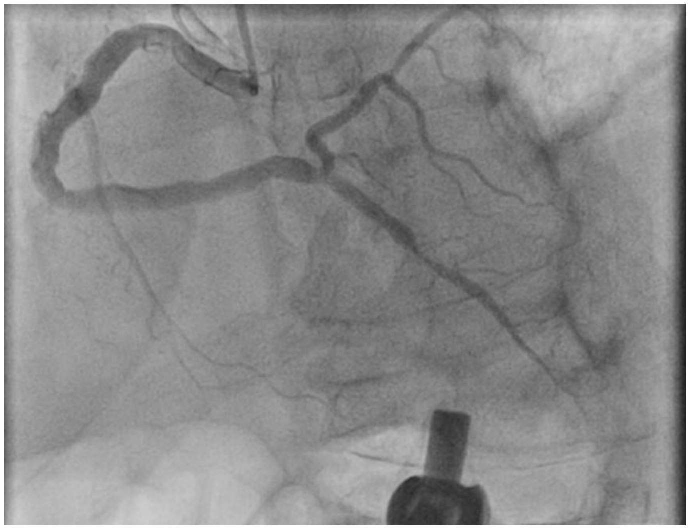

Figure S7 Patient 5

Patient 5

CCTA	ICA

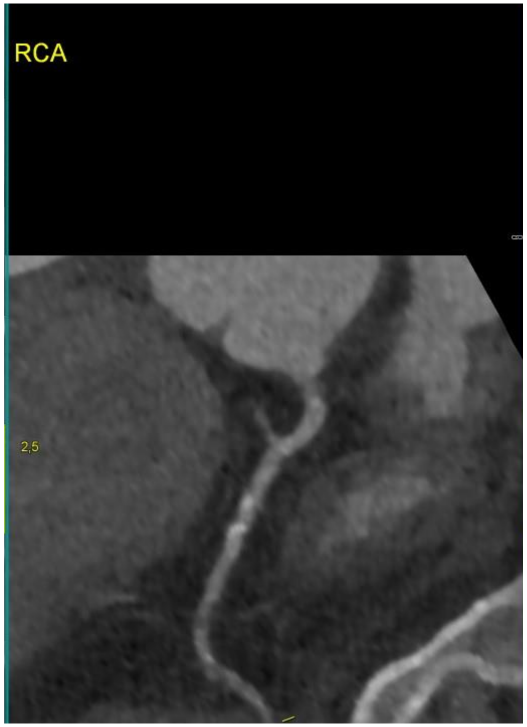

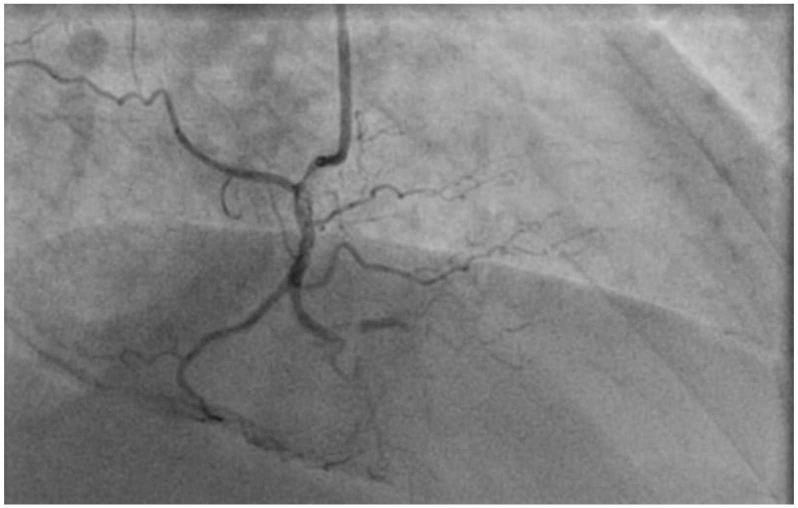

Supplement: Supplementary file 1 [file diagnostics-15-00890-s001.zip › diagnostics-3519506-supplementary.pdf]
